# Determination of the genetic structure of remnant *Morus boninensis *Koidz. trees to establish a conservation program on the Bonin Islands, Japan

**DOI:** 10.1186/1472-6785-6-14

**Published:** 2006-10-11

**Authors:** Naoki Tani, Hiroshi Yoshimaru, Takayuki Kawahara, Yoshio Hoshi, Fuyuo Nobushima, Takaya Yasui

**Affiliations:** 1 Department of Forest Genetics, Forestry and Forest Products Research Institute, Matsunosato, Tsukuba, Ibaraki 305–8687 Japan; 2 Hokkaido Research Center, Forestry and Forest Products Research Institute, Hitsujigaoka, Sapporo 062–8516 Japan; 3 Non-profit Organization for Research of Wild Organisms in Ogasawara, Chichi-jima, Ogasawara, Tokyo 100–2101 Japan

## Abstract

**Background:**

*Morus boninensis*, is an endemic plant of the Bonin (Ogasawara) Islands of Japan and is categorized as "critically endangered" in the Japanese red data book. However, little information is available about its ecological, evolutionary and genetic status, despite the urgent need for guidelines for the conservation of the species. Therefore, we adopted Moritz's MU concept, based on the species' current genetic structure, to define management units and to select mother tree candidates for seed orchards.

**Results:**

Nearly all individuals of the species were genotyped on the basis of seven microsatellite markers. Genetic diversity levels in putative natural populations were higher than in putative man-made populations with the exception of those on Otouto-jima Island. This is because a limited number of maternal trees are likely to have been used for seed collection to establish the man-made populations. A model-based clustering analysis clearly distinguished individuals into nine clusters, with a large difference in genetic composition between the population on Otouto-jima Island, the putative natural populations and the putative man-made populations. The Otouto-jima population appeared to be genetically differentiated from the others; a finding that was also supported by pairwise *F*_ST _and *R*_ST _analysis. Although multiple clusters were detected in the putative man-made populations, the pattern of genetic diversity was monotonous in comparison to the natural populations.

**Conclusion:**

The genotyping by microsatellite markers revealed strong genetic structures. Typically, artificial propagation of this species has ignored the genetic structure, relying only on seeds from Otouto-jima for replanting on other islands, because of a problem with inter-specific hybridization on Chichi-jima and Haha-jima Islands. However, this study demonstrates that we should be taking into consideration the genetic structure of the species when designing a propagation program for the conservation of this species.

## Background

*Morus boninensis*, a plant native to the Bonin Islands (typical oceanic islands, located 1,000 km south of Tokyo, Japan), is only endemic to Otouto-jima, Chichi-jima and Haha-jima Islands; it is categorized as "critically endangered" in the Japanese Red Data Book [1]. This species is a typical case in which there is little information about the species, although guidelines are urgently needed to aid in its conservation. There are fewer than about 170 remaining trees and natural regeneration does not seem to be occurring at present (Yoshimaru et al. unpublished data). The reason for the degradation of the species was intensive logging during the last quarter of the 19th century and the start of the 20th century (details described in [2]). Although *Morus boninensis *used to be one of the main species constituting the canopy in the moist tall forest on the Bonin Islands, some invasive trees, mainly *Bischofia javanica*, have replaced it in recent years [3-5]. In our field observations, seedling recruitment has not been observed since 1995. Yoshimaru et al. (unpublished data) estimated that the mortality rate of the mature individuals is between 0.56% and 3.56% per year in each population. Furthermore, hybridization with the introduced species, *M. acidosa*, has been observed and has been confirmed by molecular marker analysis [2]. To promote the propagation of the next generation, selection of mother trees should be considered to maximize evolutionary success based on the concept of the Evolutionary Significant Unit (ESU, [6]). To achieve this, it is best practice to define ESUs based on genetic as well as ecological information. However, there is no ecological information about the species. Furthermore, the Bonin Islands are a typical example of the changing balance in Japan between bio-diversity and single-minded development, between the desire to conserve native species and the desire to satisfy human desires, and between the modesty and creativity of local peoples and the arrogance and insensitivity inherent in massive public works funding[7]. Therefore, it is urgent that guidelines for conducting *ex situ *conservation and promoting the propagation of individuals for the next generation are put in place. One proposal by Moritz [8] was that the population ESU should be defined by the reciprocal monophyletic relationship based on mtDNA alleles and significant divergence of allele frequencies at nuclear loci (Moritz's Management unit, MU). Although Crandall et al [6] identified several conceptual and practical problems with the effectiveness of the use of a historical population structure, as defined by molecular genetic techniques, the concept has been adopted in various applied studies of animals to define conservation units based on ESUs [9-11]. Because of the pressing nature of our work, we have adopted Moritz's MU concept to define management units and aid in the selection of mother tree candidates for the seed orchards. This is based on the current genetic structure, since only genetic information is available at present. In this paper, we present a description of the current genetic structure of the species, genetic differentiation between populations and kinship within clustered individuals based on microsatellite markers. These data can be used to establish a conservation program for the species.

## Results

### Genetic variation within the operational populations

**Figure 1:**
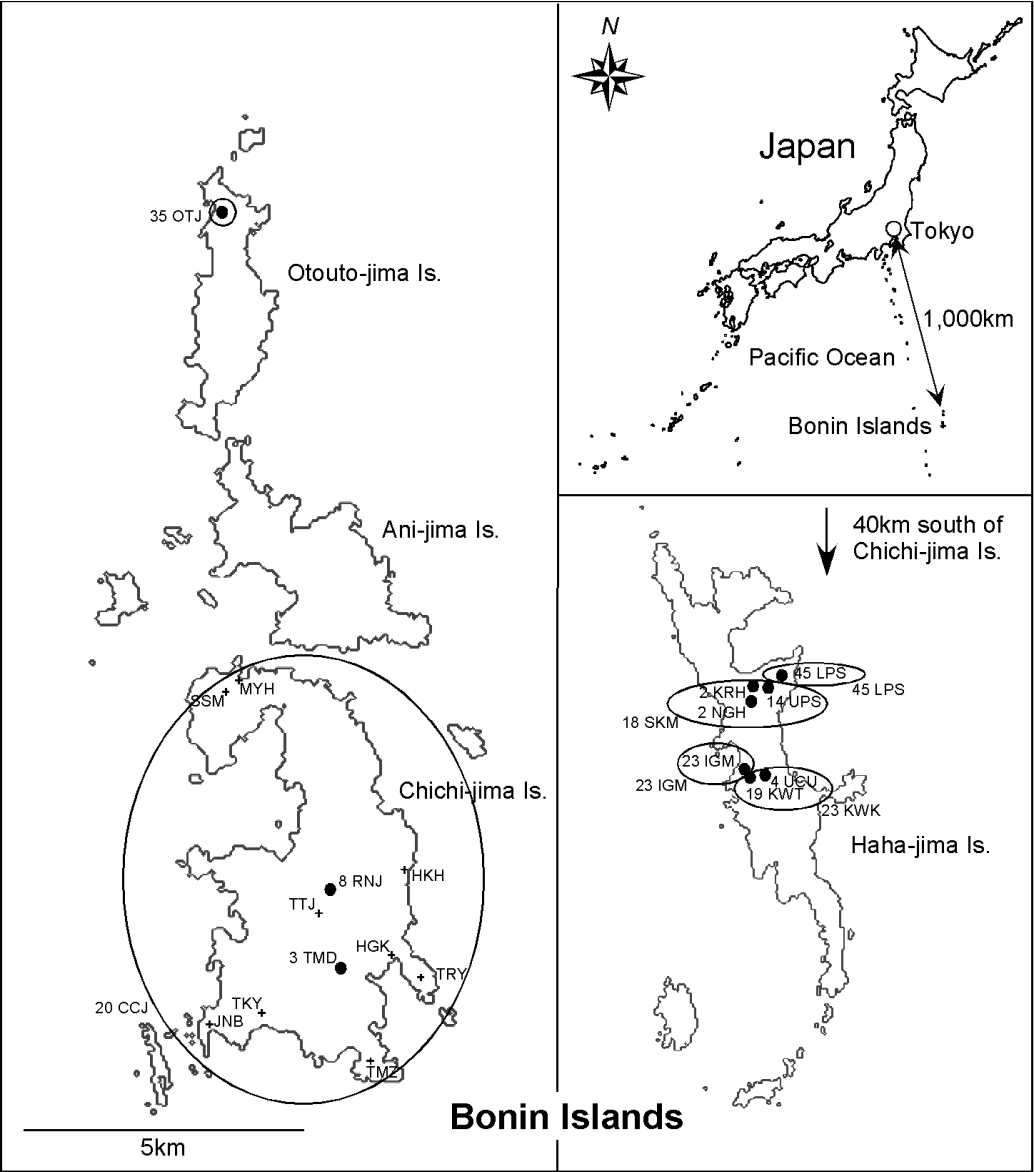
Location of the Bonin Islands and the location of each remnant tree of *M. boninensis*. The plus signs and black dots represent solitary trees and tree clumps, respectively. The number beside each black dot indicates the number of trees in that clump. The ellipses indicate the six operational populations.

**Figure 2:**
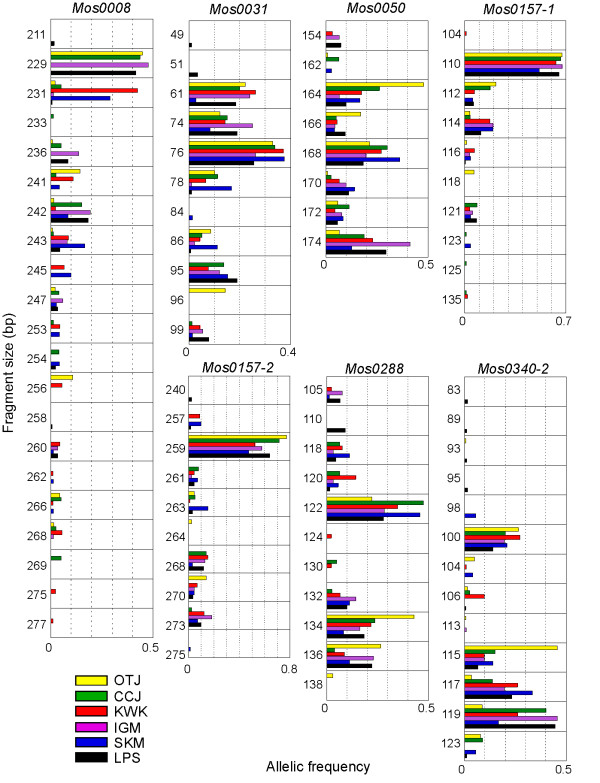
The distribution of alleles of each population for the seven microsatellite loci.

In total, 164 remnant trees were genotyped (data from two trees were missing). Based on their geographic distribution, these individuals were assigned to one of the six operational populations (Table 1, Fig. 1). Maximum (21) and minimum (8) numbers of alleles were detected at *Mos0008 *and *Mos0050 *loci, respectively. Although alleles with the highest frequency were common between the operational populations at three loci, the other four loci did not share the highest frequency alleles between the operational populations (Fig. 2). The genetic variation within populations indicated by population genetic statistics that are not affected by population size, such as allelic richness (*A*_i_), observed and expected heterozygosity (*H*_o _and *H*_e_), are responsible for the highest values in the KWK and SKM populations (Table 2). In contrast, the CCJ population exhibited the smallest amount of within population genetic variation when these statistics were considered. The difference between the largest and smallest amounts of genetic variation was, however, not large, indicating that these operational populations retain a similar level of genetic variation with some fluctuation. We observed significant deviation between the fixation indices of the CCJ, IGM and LPS populations when double reduction was not considered. An excess of homozygotes was observed at two loci in the CCJ population. The IGM and LPS populations exhibited an excess of heterozygotes at all significant loci, with the exception of *Mos0288 *in the LPS population. All statistically significant deviation of the fixation indices indicates heterozygote excess if we assume that maximum double reduction occurred (Table 3).

### Genetic differentiation among the operational populations

**Figure 3:**
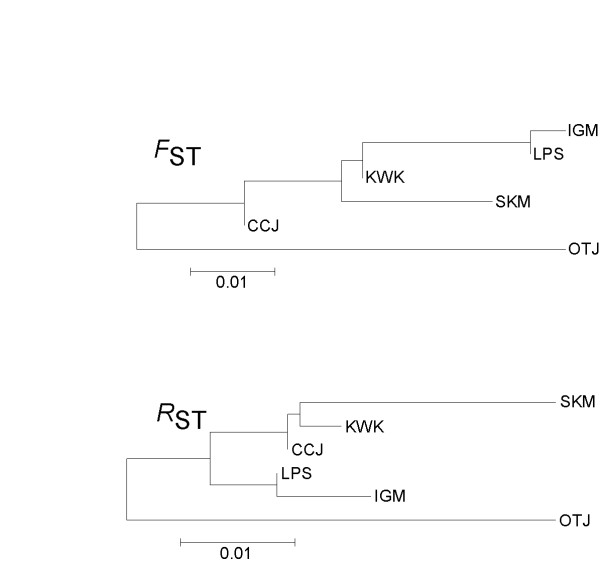
Neighbour-joining trees based on pair-wise *F*_ST _and *R*_ST _among operational populations of *M. boninensis*.

Global *F*-statistics indicate that there is highly significant population differentiation between populations at all microsatellite loci. However, Global *R*-statistics based on the stepwise mutation model showed that significant population differentiation occurred at five loci, but not at the two others. The significance level was much smaller in three of the five significant loci than those of *F*_ST _(Table 4). The genetic relationship between populations was determined by constructing a neighbour-joining tree based on pairwise *F*_ST _and *R*_ST_(Fig. 3). There was no difference in the topologies of the two phenograms, however, branch length was different. OTJ was the population most genetically separated from the others; this was supported by both pairwise *F*_ST _and *R*_ST_. Both phenograms showed that IGM and LPS had similar genetic compositions. The branch length between KWK and CCJ was shorter in the phenogram based on *R*_ST_, than in the one based on *F*_ST_. It seems that KWK has a similar genetic composition to CCJ. However, SKM's genetic composition was different from KWK, as indicated by the long branch associated with SKM that diverges close to KWK in both the *F*_ST _and *R*_ST _phenograms.

### Individual based analysis using the model-based clustering method

**Figure 4:**
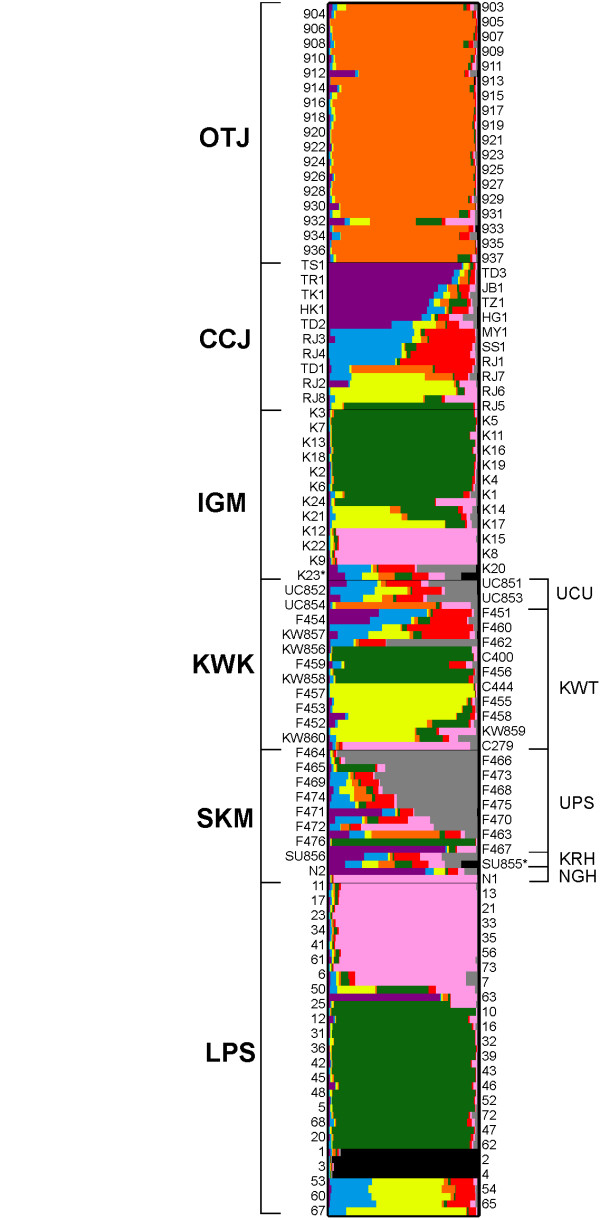
Bar plots of estimates of membership coefficient (Q) for each individual for the inferred clusters (*K *= 9) with maximum log-likelihood probability. Bar colours and lengths represent inferred clusters and Q, respectively.

We performed model-based clustering in order to elucidate the population structure; we used multilocus microsatellite genotypes, but excluded *Mos0157-2*, as it is closely linked to *Mos0157-1*. A model that considered both admixture and uncorrelated allele frequency was adopted for the analysis. Our reasoning was that we had observed many stumps of *M. boninensis *and the species exhibits many characteristics, such as dioecism and anemophily, which might account for the admixture believed to have occurred in the past [12]. The highest posterior probability was obtained for nine clusters (*K *= 9). All individuals growing in the OTJ operational population were assigned to a single cluster (represented in orange in Fig. 4), which was supported by 90% probability intervals for all but two individuals (912 and 932). Although in the IGM and LPS populations individuals were mainly assigned into three and four clusters, respectively, most individuals belonged to two clusters represented by green and pink in the diagram. As well as the two main clusters, the LPS operational population contained four and five individuals, respectively, belonging to the black and yellow clusters. The individuals in the CCJ population were predominantly assigned into three clusters. The trees growing in the south part of Chichi-jima Island belonged to one particular cluster (purple), with the exception of TD1 and RJ1-8. Eight trees in RJ were separately assigned to three different clusters, rather than the cluster represented by purple (Fig. 4). Two individuals from the northern part of Chichi-jima Island were assigned to a cluster containing three of the RJ individuals (shown in blue and red in Fig. 4). We observed a more complicated pattern of genetic composition, as determined by the clustering analysis, in the KWK and SKM operational populations of Haha-jima Island. Three main types of genetic composition were observed in the KWK population. Two of these were simply expressed by the green and yellow colours in Fig. 4. The genetic composition of another type was composed of three or four clusters (mainly grey, red, blue and purple) with more or less equal probability. The genetic composition of this type was similar to that of UPS in the SKM operational population. However, individuals in the UPS population were classified as belonging to the grey cluster at a higher probability than the individuals in KWK (Fig. 4).

### Genetic diversity and kinship structure within the clusters

**Figure 5:**
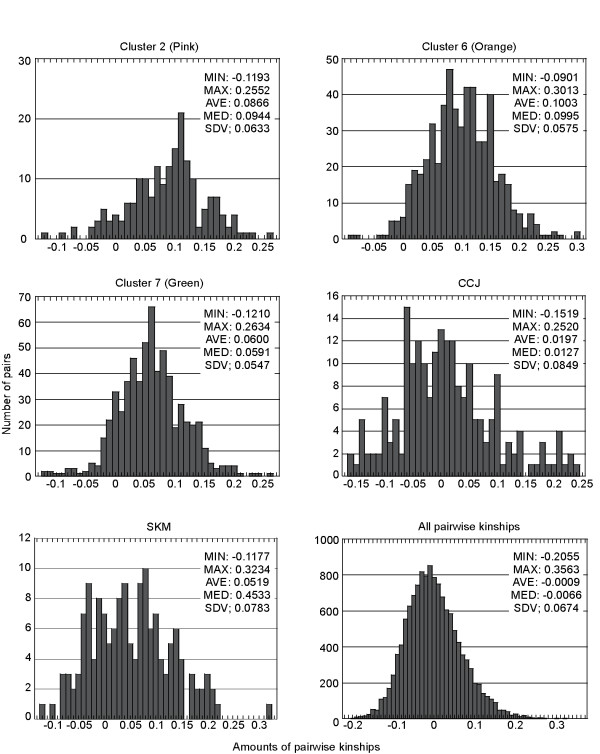
Distribution of the pairwise kinship coefficient between individuals supported by 90% probability intervals of Q within each cluster. Clusters 2, 6 and 7 correspond to the pink, orange and green clusters shown in Figure 4. MIN, MAX, AVE, MED and SDV represent minimum value, maximum value, mean, median and standard deviation, respectively, for each dataset.

When we investigated whether attribution of individuals to the clusters was supported by a 90% confidential interval, 65 individuals could not be classified to a particular cluster at this level. The remaining 99 individuals were each classified to one of seven clusters with 90% certainty. Because four of these clusters did not contain enough individuals, we estimated the pairwise kinship values for only three clusters. Cluster 2 (pink in Fig. 4) was composed mostly of individuals from LPS, plus five individuals from IGM and two from KWT and NGH. Cluster 6 (orange in Fig. 4) comprised all the individuals in the OTJ population, although two of them were not supported by the 90% confidence interval. Cluster 7 (green in Fig. 4) contained 19 individuals from LPS, 11 individuals from IGM, four individuals from KWT and a single individual from UPS. The amounts of genetic diversity were estimated for these clusters. In general, there were fewer alleles within the clusters than within the operational populations. However, the clusters retained almost the same levels of the observed heterozygosity as found in the operational populations. We estimated pairwise kinship between individuals of each of the clusters, two operational populations (CCJ; mixed background of the population and SKM; putative natural of it) and all individuals using Loiselle et al.'s formula [13]. The kinships between all individuals were normally distributed, with a mean and standard deviation of nearly zero and 0.0674, respectively. Although the pairwise kinships of three clusters were almost normally distributed, their means were significantly different from zero. Maximum and minimum values of the means were 0.1003 and 0.0600 in clusters 6 and 7, respectively. Although numbers of pairs for CCJ and SKM were relatively fewer than those of the clusters, their means were not significantly different from zero (Fig. 5).

## Discussion

### Genetic diversity within populations

The model-based clustering method demonstrated that the genetic structure of the *M. boninensis *remnant trees did not correspond perfectly to the operational populations based on geographical distribution (Fig. 4). Although the OTJ operational population did coincide with the model based clustering, other operational populations contained multiple clusters. However, in determining operational populations to be used in species conservation, there are two considerations of practical importance: 1) the convenience of conducting controlled crossing using trees in close physical proximity, whilst knowing their individual genetic relatedness; and 2) the ability to sample seed that may have been open pollinated from nearby paternal trees. Therefore, it is important to understand the genetic composition at two levels: the operational population and the individual level. The genetic diversity levels in OTJ and CCJ were lower than in the other populations (Table 2). Although the OTJ population contains the secondly largest number of trees of all the operational populations, the genetic composition of each tree is very similar, as demonstrated by the model-based clustering (Fig. 4). This might be the result of a founding event, with a small number of maternal trees having produced the seed to establish the extant OTJ population. However, the low genetic diversity in the CCJ population is different. Four different genetic groups were identified in the CCJ population by model-based clustering analysis. Out of the four genetic groups, some of the trees comprising three of them (blue/red, yellow and green in Fig 4) are located in the northern part of Chichi-jima Island. This is an area inhabited by immigrant people. Trees in these same genetic groups were also present in KWK on Haha-jima Island. Therefore, these trees in CCJ were probably planted by people using seed sources from Haha-jima Island. In contrast, the vegetation of the south of Chichi-jima is, to date, well preserved although there was some disturbance during World War II. These trees have retained their endemic genetic composition (purple in Fig. 4) on Chichi-jima Island, which probably represents the native genetic composition of the species here. We only found nine remnant trees classified with the purple genetic composition in the CCJ population. Although the expected heterozygosity level of the nine remnant trees was smaller than that of CCJ (0.628 vs. 0.674), their allelic richness exceeded that of CCJ (2.476 vs. 2.429). This might be because a limited number of maternal trees were used as the seed source for planting the trees in the northern part of Chichi-jima Island, or because of bi-parental inbreeding of parent trees of this population.

On Haha-jima Island, we observed a similar pattern of genetic diversity as for the CCJ population. Trees on Haha-jima Island were divided into four operational populations. One of the current authors (Y. Hoshi) remembered that the species had been grown in plantations on this Island. He recollected that part of KWK and LPS were planted by humans. Although the origin of the IGM population was not clear, the small, relatively uniform trees in IGM are distributed in quite a small area. This might indicate that, whilst these trees were not planted, they are the result of simultaneous regeneration from a few maternal trees, such as those of an original plantation. The amounts of within population genetic diversity of plantation trees (LPS) and IGM were lower than those of SKM and KWK. According to the model-based clustering, we identified a similar pattern of genetic composition between the planted populations (part of KWK and LPS) and IGM – the clusters represented by mostly green and pink colours (Fig. 4). This might also indicate an origin from a limited number of maternal trees or bi-parental inbreeding. In contrast, the rest of the trees in the KWK and SKM populations exhibited a complicated genetic composition, and the SKM population contained the highest amount of genetic diversity of all the operational populations, despite having the smallest sample size. In terms of genetic diversity and size of tree (data not shown), most trees in the SKM population and part of the KWK population are likely to be natural remnants.

### The evidence of population bottlenecks and inbreeding

The individuals in the SKM population exhibited many components of different clusters (Fig. 4). As a result of its genetic characteristics, size of trees and vegetation pattern on the Islands, we deduced that this population is the closest to being natural. When we compared the genetic diversity level between the OTJ (almost equivalent to cluster 6) and SKM populations, the number of alleles, as well as the observed and expected heterozygosity was lower in the former. According to one theoretical study, the amount of reduction in heterozygosity depends not only on the "bottleneck" size but also on the rate of population growth after passing through the bottleneck, while the loss of alleles largely depends on the size of the "bottleneck" [14]. If the SKM population produced many generations after the bottleneck event, the heterozygosity level would be reduced. However, we have assumed that the SKM population produced few generations after population decline, because *M. boninensis *is known to be an extremely long-lived woody species [12]. Although the SKM population has maintained a high level of genetic diversity to date, the decline in genetic diversity in OTJ might be the result of bottleneck events, such as much severe logging, long-term population decline, or catastrophic population destruction.

When we estimated the pairwise kinships between individuals within a cluster and compared their average between clusters, the difference in average pairwise kinships between cluster 6 and all individuals was significant (Fig. 5). This meant that bi-parental inbreeding may have occurred during the bottleneck event in the OTJ population. However, the level of inbreeding was, supposedly, not strong, although expression of strong inbreeding depression for the species may have been masked by mass mortality of inbred individuals at the seedling stage. This speculation is also supported by the non-significant and negative fixation indices in cluster 6 and OTJ (Table 3). In contrast, potentially man-made populations, such as parts of KWK and LPS, and IGM, contained multiple clusters. However, most individuals within these populations were classified into two main clusters (green and pink), supported by the 90% confidence interval in the model-based clustering analysis (Fig. 4). Furthermore, we detected higher pairwise kinship values within clusters (green and pink) than those for all pairs of individuals (Fig. 5). This could be the consequence of a limited number of maternal trees producing the seeds from which the man-made populations were propagated. In contrast, although the mean of the pairwise kinship of SKM was somewhat high value, the mean of pairwise kinship of the putative natural populations and mixed background population, SKM and CCJ, is not significantly different from zero. This suggests that these populations might not have experienced intensive bi-parental inbreeding, as occurred in the putative man-made populations. If we collect seed from a few mother trees for the propagation of the next generation, even open pollination will potentially cause bi-parental inbreeding and a decline in the genetic diversity of the new generation. Therefore, it is very important to construct seed orchards using clones of trees from natural populations and/or to perform controlled crossing between them. Furthermore, some pairs in SKM population represented high level of pairwise kinships between them, which implied that we must consider selection of parental trees to construct seed orchards and combinations of parental trees for control crossing even if parental trees are only selected from the natural populations

### Establishment of conservation units and a propagation program

*Ex situ *conservation would seem to be necessary to conserve *M. boninensis*, because it is difficult to obtain seeds free of hybridization with *M. accidosa*. The exception is the OTJ population [2]. In addition, seedling establishment is extremely rare because of the competition with introduced alien species, such as *Bischofia javanica *[5]. Therefore, selection of pure *M. boninensis *seedlings, controlled crossing and *ex situ *conservation are all necessary to ensure propagation of the species. To achieve this, we must consider Moritz's management units in order to select appropriate mother trees and we must undertake controlled crossing to obtain seed sources for propagation. In terms of genetic diversity, there was a large difference between the putative natural populations, with the exception of OTJ and IGM (south of CCJ, a part of KWK, most of SKM) and putative plantation trees. According to the model-based clustering analysis, planted individuals are classified into two main clusters, green and pink. This genetic differentiation, which is supported by the model-based clustering, should be used in selecting the MUs. We were able to identify at least seven main MUs for *M. boninensis*, which were OTJ, natural trees in CCJ, natural trees in KWK, SKM, man-made trees in CCJ and KWK and two major clusters including individuals in IGM and LPS (Green and Pink). Natural populations retained many elements of the different clusters. On the other hand, most of the plantation trees were simply classified into a single cluster. Hence, natural individuals were prime candidates for use as maternal trees in controlled crossing and *ex situ *conservation by grafting. According to analyses of pairwise *F*_ST _and *R*_ST _and the model-based clustering, we should avoid controlled crossing and instead should establish new seed orchards by open pollination of individuals from different clusters. To date, the OTJ population has been the only one to produce pure *M. boninensis *seeds. These seeds, therefore, have been the only ones used for propagation. There is a hybridization problem with the introduced species in the KWK and SKM populations. Because the MU of OTJ is definitely different from the other natural populations, we need to independently conduct controlled crosses or *ex situ *conservation (and subsequently develop seed orchards) within each cluster. Only then will propagated seedlings be available to use for propagation of the species in the forest according to the MUs identified in this study.

## Conclusion

Genotyping using microsatellite markers revealed that the pattern of genetic variation was different between the OTJ population, the north CCJ putative man-made population, the south CCJ putative natural population, the natural population in Haha-jima (most of SKM and a part of KWK), IGM and the man-made population in Haha-jima (a part of KWK and LPS). These differences should guide the selection of the MUs. The putative natural populations exhibited higher genetic diversity than those of the man-made populations, IGM and OTJ. These are, therefore, important genetic resources for the propagation of a new generation. To date, propagation of the species has ignored its genetic structure by relying only on seeds from the OTJ population, even for planting on the other islands. This has been because of the inter-specific hybridization problem on Chichi-jima and Haha-jima Islands. However, this study has demonstrated the high level of genetic differentiation within the species. This needs to be considered with respect to any propagation program aimed at conserving the evolutionary range of the species.

## Methods

### Field survey and collection of samples

Between 1998 and 2002, we conducted a field survey to find the unknown remnant trees of *M. boninensis*. Summary of field survey concerning with background of each population, location, tree size and vegetation was shown in Table 1. Identification of true *M. boninensis *or hybrids between *M. boninensis *and *M. acidosa *was conducted, following sampling, using the three SCAR markers [2]. This allowed us to exclude hybrids from subsequent population genetic analyses. To date, we have found and collected petioles from 35 trees on Otouto-jima Island (OTJ), 20 trees on Chichi-jima Island (CCJ) and 109 trees on Haha-jima Island (Yoshimaru et al. unpublished data). Four areas were recognized in terms of their geography and the distributional density of the remnant trees on Haha-jima Island. Therefore, we assumed that these four geographical areas would be operational populations. We gave them the names: Iguma-wan (IGM), Kuwanoki-yama (KWK), Sekimon (SKM) and lower plateau of Sekimon (LPS). We were able to recognize some sub-groups of trees within two operational populations: KWK and SKM. KWK contained Uchu-sawa (UCU) and Kuwanoki-yama test field (KWT). SKM contained the upper plateau of Sekimon (UPS), Kiri-hama (KRH) and Naga-hama (NGH). However, we did not deal with these tree clusters as operational populations because they contained relatively few individuals. Thus, 23, 23, 18 and 45 remnant trees were identified and sampled in the IGM, KWK, SKM and LPS operational populations, respectively. The petioles from these trees were stored at -20°C until the DNA was extracted.

### DNA preparation and genotyping of microsatellite loci

DNA was extracted from 100 mg of the petioles of each of the sampled remnant trees using a DNeasy plant-mini Kit (QIAGEN, Hilden, Germany) and following the manufacturer's instructions. The genomic DNA concentration was adjusted to 5 ng/μL by dilution after measuring the concentration of extracted DNA using a spectrophotometer (Amersham Biosciences, Little Chalfont, UK). For all trees, the genotypes of seven microsatellite markers were assigned according to the procedure described by Tani et al. [15]. Because *M. boninensis *is a putative autotetraploid species, we determined the allele copy number of partial heterozygotes based on dosage of electrophoretogram peaks.

### Operational populations based analyses

We based the composition of the six operational populations on geographical distribution and individual tree aggregations. For these we calculated population genetic statistics as follows: the number of alleles (*A*); the average number of alleles per individual (*A*_i_); the average number of four allele genotypes (*G*); and observed heterozygosity (*H*_o_). These were obtained for each locus using the program AUTOTET [16]. Two types of expected heterozygosities (*H*_e_) and fixation indices (*F*_is_) were calculated under two assumptions: 1) random mating and random chromosome segregation (RceS); and 2) random mating and some level of chromatid segregation (RcdS), for which we selected the maximum theoretical double reduction rate, α = 1/7 [17-19]. Using SPAGeDi software, *F*-statistics and *R*-statistics, based on the infinite allele model and the stepwise mutation model, respectively, were estimated in order to evaluate genetic diversity between the operational populations [20-22]. Random resampling of individuals was parmuteted at 20,000 times to obtain a confidence interval for the estimators of population differentiation and the inbreeding coefficient. The genetic relationships between the operational populations were elucidated using neighbor-joining trees based on pairwise *F*_ST _and *R*_ST_, with the aid of two software packages, Mega ver. 2.1 and SPAGeDi [20,23,24].

### Individual based analyses

To identify any unknown population structure, we used a model-based clustering method implemented by the program Structure ver. 2.0. This estimated the number (*K*) of clusters into which the sample data (*X*) were fitted with posterior probability Pr(*X*|*K*), using a model with admixture and uncorrelated allele frequency [25]. We conducted 10^6 ^iterations following a burn-in period of at least 30,000 iterations. The genotypes of the *Mos0157-2 *locus were omitted from the data set because *Mos0157-2 *was constructed from same sequence as *Mos0157-1*, and hence these loci were closely linked to each other. *K *provides only a rough guide for determining which models are consistent with the data. Therefore, we examined various *K *values, from 1 to 20, within the simulation, and searched for a suitable *K *value to maximise the posterior probability Pr(*X*|*K*). The Structure software estimates the proportion of ancestry (*Q *value) from each of the *K *clusters for each individual and the 90% probability intervals. Assignment of individuals into inferred populations was conducted using the *Q *values and their probability intervals. Only individuals supported by 90% probability intervals were assigned to each cluster. A kinship coefficient between individuals within a cluster was estimated using SPAGeDi software [13,20]. The amount of kinship within each cluster was estimated by comparing the probability of two individuals in a cluster having identical genes to the probability that two individuals chosen at random from all the samples are identical.

## Authors' contributions

NT performed the sampling, molecular analyses, data interpretation, wrote the manuscript and was responsible for this study of M. boninensis. HY performed the sampling, constructed the database for the remnant trees and interpreted the data. TK performed the sampling and data interpretation. YH, FN and TY performed the survey of the remnant trees and the sampling. All authors read and approved the final manuscript.

## Figures and Tables

**Table 1:** Information of sampling sites and trees, such as background of population, location, tree size and vegetation

	Background of the population	Latitude and Longitude		Elevation	Population density
OTJ	putative natural	N27° 10'46"–10'53"	E142° 11'18"–11'24"	33–90 m	Dense
CCJ	mixed^1)^	N27° 02'40"–06'14"	E142° 11'07"–14'00"	2–300 m	Sparse
KWK	mixed^2)^	N26° 39'32"–39'50"	E142° 08'59"–09'20"	188–334 m	Intermediate
IGM	unknown	N26° 39'38"–39'40"	E142° 08'58"–09'00"	155–172 m	Dense
SKM	putative natural	N26° 40'08"–40'25"	E142° 09'08"–08'37"	200–314 m	Sparse
LPS	putative man-made	N26° 40'52"–40'57"	E142° 09'48"–09'53"	174–183 m	Dense

	Tree size (DBH)	Vegetation			

OTJ	15–77 cm	Natural			
CCJ	11–84 cm	Various type of vegetation			
KWK	17–95 cm	Introduced species dominating			
IGM	17–52 cm	Putative natural			
SKM	20–119 cm	Natural			
LPS	16–49 m	Second growth			

^1)^The planted trees were sampled in northern civilization area and the middle part of the island, trees in the south part of the island supposed to be natural.

^2)^The planted trees are located in the lower elevation (to ca. 240 m), the natural trees are supposed to grow in the higher elevation area.

**Table 2:** Average genetic variation of each locus for five operational populations of *Morus boninensis*. Standard deviation of the mean is in parenthesis. Clusters 2, 6 and 7 are groups assigned by the model-based clustering analysis with a 90% confidence interval

					Chromosome (Ce)	Chromatid (Cd)
					
Pop	*N*	*A*	*Ai*	*H*_o_	*H*_e _(Ce)	*H*_e _(Cd)
OTJ	35	6.429 (2.760)	2.523 (0.447)	0.659 (0.161)	0.620 (0.151)	0.579 (0.141)
CCJ	20	7.286 (2.752)	2.429 (0.346)	0.630 (0.129)	0.674 (0.136)	0.629 (0.127)
KWK	23	8.000 (2.380)	2.758 (0.252)	0.732 (0.085)	0.731 (0.084)	0.682 (0.079)
IGM	23	6.571 (1.618)	2.708 (0.451)	0.712 (0.128)	0.681 (0.117)	0.636 (0.109)
SKM	18	7.571 (1.718)	2.701 (0.187)	0.712 (0.057)	0.735 (0.065)	0.686 (0.060)
LPS	45	8.143 (2.035)	2.589 (0.520)	0.669 (0.166)	0.661 (0.137)	0.645 (0.127)

Cluster 2	19	4.571 (0.976)	2.709 (0.625)	0.710 (0.207)	0.639 (0.158)	0.597 (0.147)
Cluster 6	33	6.000 (2.582)	2.524 (0.440)	0.661 (0.158)	0.616 (0.146)	0.575 (0.136)
Cluster 7	35	4.714 (0.756)	2.666 (0.583)	0.695 (0.186)	0.634 (0.155)	0.592 (0.145)

**Table 3:** Fixation indices for five operational populations of *Morus boninensis*, with and without double reduction. Clusters 2, 6 and 7 are groups assigned by the model-based clustering analysis with a 90% confidence interval

	*F *(Chromosome, Ce)								
	
Locus	OTJ	CCJ	KWK	IGM	SKM	LPS	Cluster 2	Cluster 6	Cluster 7
*Mos0008*	-0.037	0.178*^1^	0.001	-0.027	0.144	0.107	-0.090	-0.043	-0.049
*Mos0031*	-0.006	-0.003	0.029	-0.034	-0.029	-0.001	-0.104	-0.020	-0.125
*Mos0050*	-0.128	0.152*	0.049	-0.066	0.023	-0.082*	-0.157*	-0.137	-0.125*
*Mos157-1*	-0.043	-0.024	-0.018	-0.042	-0.046	0.070	-0.077	-0.048	-0.072
*Mos157-2*	-0.077	0.113	-0.012	-0.102	0.100	0.129	0.066	-0.077	-0.110*
*Mos0288*	-0.043	-0.041	-0.060	-0.091**	-0.006	0.046***	-0.164*	-0.060	-0.138***
*Mos340-2*	-0.113	0.067	-0.002	0.044	0.018	0.012	-0.139*	-0.118	-0.015

Average	-0.063	0.066	-1.000	-0.046	0.032	0.032	-0.110	-0.072	-0.095
S. D.	0.032	0.063	0.027	0.034	0.052	0.048	0.052	0.031	0.039

	*F *(Chromatid, Cd)								
	
Locus	OTJ	CCJ	KWK	IGM	SKM	LPS	Cluster 2	Cluster 6	Cluster 7

*Mos0008*	-0.111	0.119	-0.071	-0.101	0.082	0.043	-0.168	-0.118	-0.123
*Mos0031*	-0.078	-0.075	-0.040	-0.108	-0.103	-0.073	-0.183*	-0.093	-0.205***
*Mos0050*	-0.208*	0.092	-0.019	-0.143	-0.046	-0.160***	-0.240***	-0.218*	-0.205***
*Mos157-1*	-0.117	-0.097	-0.091	-0.117	-0.121*	0.003	-0.154	-0.123	-0.148
*Mos157-2*	-0.154	0.050	-0.084	-0.181	0.036	0.067	-0.001	-0.154	-0.189**
*Mos0288*	-0.117	-0.116	-0.136*	-0.168***	-0.078	-0.022**	-0.247**	-0.136	-0.219***
*Mos340-2*	-0.193*	0.000	-0.073	-0.025	-0.052	-0.058	-0.221**	-0.198*	-0.087

Average	-0.139	-0.001	-0.073	-0.120	-0.037	-0.037	-0.189	-0.149	-0.174
S. D.	0.035	0.061	0.027	0.036	0.050	0.054	0.062	0.035	0.047

^1^Asterisks indicate statistical significance of fixation index at (*) 5%, (**) 1% and (***) 0.1%, based on goodness of fit between observed and expected genotype frequencies.

**Table 4:** The global *F *and *R *statistics for seven microsatellite loci

	*F*_IT_	*F*_IS_	*F*_ST_	*R*_IT_	*R*_IS_	*R*_ST_
All loci	0.0558***	0.0092	0.0470***	0.0924***	0.0645***	0.0298***
*Mos0008*	0.0873***	0.0653***	0.0235***	0.0507	0.0328	0.0185*
*Mos0031*	0.0282*	0.0025	0.0258***	0.1151***	0.1077**	0.0083
*Mos0050*	0.0401**	-0.0260*	0.0644***	-0.0188	-0.0878**	0.0634***
*Mos0157-1*	0.0232	-0.0025	0.0256***	0.0319	0.0268	0.0052
*Mos0157-2*	0.0798***	0.0407*	0.0408***	0.0632*	0.0373	0.0268**
*Mos0288*	0.0378**	-0.0139	0.0510***	0.1368***	0.0436	0.0975***
*Mos0340-2*	0.0931***	0.0065	0.0872***	0.1375***	0.1208***	0.0190*

^1^Asterisks indicate statistical significance at (*) 5%, (**) 1% and (***) 0.1%, based on permutation test at 20,000 times
